# Supplementary material for: Factors associated with iron deficiency anaemia among pregnant teenagers in Ashanti Region, Ghana: A hospital-based prospective cohort study
Source: PLoS One. 2021 Apr 27;16(4):e0250246. doi: 10.1371/journal.pone.0250246 (PMC8078754; doi:10.1371/journal.pone.0250246)
Supplement: S1 Table — (DOCX) [file pone.0250246.s001.docx]

**Supplement 1**. **Relationship between sociodemographic and haemoglobin status.**

|  |  | **Hb status** | |  |  |
| --- | --- | --- | --- | --- | --- |
| **Variables** | **Total** | **Anaemia** | **No anaemia** | **Chi-square** | **P value** |
| **Community type** |  |  |  |  |  |
| Rural | 173(41.6) | 103(60.9) | 66(39.1) | 1.711 | 0.222^ǂ^ |
| Urban | 243(58.4) | 129(54.4) | 108(45.6) |  |  |
| **Age group (years)** |  |  |  |  |  |
| 13-15 | 32 (7.7) | 16(51.6) | 15(48.4) | 0.419 | 0.573^ǂ^ |
| 16-19 | 384 (92.3) | 216(57.6) | 159(42.4) |  |  |
| **Marital status** |  |  |  |  |  |
| Single | 316 (76.0) | 183(59.4) | 125(40.6) | 2.691 | 0.103^ǂ^ |
| Married | 100 (24.0) | 49(50.0) | 49(50.0) |  |  |
| **Educational level** |  |  |  |  |  |
| None | 19 (4.6) | 10(52.6) | 9(47.4) | 0.750 | 0.861^¥^ |
| Primary | 56 (13.5) | 31(55.4) | 25(44.6) |  |  |
| JHS | 255 (61.3) | 141(56.6) | 108(43.4) |  |  |
| SHS | 86 (20.7) | 50(61.0) | 32(39.0) |  |  |
| **Occupation status** |  |  |  |  |  |
| Unemployed | 298 (71.6) | 162(55.7) | 129(44.3) | 0.910 | 0.374^ǂ^ |
| Employed | 118 (28.4) | 70(60.9) | 45(39.1) |  |  |
| **Parity** |  |  |  |  |  |
| One | 316 (76.0) | 182(59.1) | 126(40.9) | 1.977 | 0.162^ǂ^ |
| More than one | 100 (24.0) | 50(51.0) | 48(49.0) |  |  |
| **Income (Ȼ)** |  |  |  |  |  |
| None | 310 (74.5) | 169(56.1) | 132(43.9) | 1.307 | 0.727^ǂ^ |
| Below 100 | 41 (9.9) | 26(63.4) | 15(36.6) |  |  |
| Between 100- <500 | 61 (14.7) | 34(56.7) | 26(43.3) |  |  |
| Between 500-1000 | 4 (1.0) | 3(75.0) | 1(25.0) |  |  |

Data are presented as frequency (percentage)^¥^- Chi-square P value, ^ǂ^- Fisher’s exact P value
