# Supplementary material for: Factors associated with iron deficiency anaemia among pregnant teenagers in Ashanti Region, Ghana: A hospital-based prospective cohort study
Source: PLoS One. 2021 Apr 27;16(4):e0250246. doi: 10.1371/journal.pone.0250246 (PMC8078754; doi:10.1371/journal.pone.0250246)
Supplement: Attachment — Submitted filename: PONE-D-20-21782_reviewer.pdf [file pone.0250246.s005.pdf]

PLOS ONE

Factors associated with Iron Deficiency Anaemia among pregnant teenagers in Ghana

--Manuscript Draft--

Manuscript Number: PONE-D-20-21782

Article Type: Research Article

Full Title: Factors associated with Iron Deficiency Anaemia among pregna[illegible]eenagers in Ghana

Short Title: Iron Deficiency Anaemia factors associated among pregnant teenagers

Corresponding Author: Reginald Adjetey Annan, MPhil
KNUST
Kumasi, GHANA

Keywords: Iron deficiency anaemia; pregnant teenagers; associated r[illegible]

Abstract:

Background

Iron Deficiency Anaemia (IDA) is reportedly high in pregnant adults and the causes well studied. However, among pregnant teenagers, the levels and associated factors of IDA are not fully understood.

Methods

In a prospective cohort study among Ghanaian pregnant teenagers, aged 13-19 years, IDA prevalence and associated factors were investigated. Sociodemographic data, household hunger scale, lived poverty index, eating behaviour, and interventions received during antenatal care (ANC) were ob[illegible]ed from 416 pregnant adolescents in Ashanti Region, Ghana. Anthropometric and n[illegible]ents intake using a repeated 24-hour dietary recall were determined and blood samples analysed for haemoglobin (Hb), serum levels of ferritin, prealbumin, vitamin A, total antioxidant capacity (TAC), C-reactive protein (CRP), and zinc protoporphyrin (ZPP).

Results

Anaemia (Hb cutoff <11.0 g/dL) was 57.1%, deficient systemic supply of iron stores (31.4%), depleted body stores of iron (4.4%), inadequate dietary iron intake (97.6%), and inadequate multiple micronutrient intakes (52.2%) were notable among study participants. Between-subject effects using Generalized Linear Modelling indicated malaria tablet given at ANC (p=0.035), MUAC (p=0.043), ZPP (p<0.001), ZPP/Hb ratio (p<0.001) and depleted body iron stores (DBIS) (p<0.001) to significantly affect Hb levels. Pregnant teenagers with a high ZPP/Hb ratio (OR= 9.7, p<0.001, 95%CI= 6.0-15.8) had an increased odds of being anaemic compared to those with normal ZPP/Hb ratio. Participants who were wasted (OR= 1.2, p = 0.543, 95%CI= 0.6-2.3), those with depleted iron stores (OR= 3.0, p = 0.167, 95%CI= 0.6-14.6) had an increased odds of being anaemic. Participants who experienced hunger (OR= 2.9, p = 0.040, 95%CI= 1.1-7.8) had an increased odd of depleted iron stores compared to those who did not experience hunger. Participants with inadequate multiple micronutrients intakes (OR= 2.6, p = 0.102, 95%CI= 0.8-8.4), and those with low serum levels of ferritin (OR= 3.3, p = 0.291, 95%CI= 0.4-29.2) had increased odds of depleted body iron stores.

Conclusions

IDA is common among pregnant teenagers and the related factors include malaria tablets given at ANC, maternal hunger, maternal MUAC, deficient systemic supply of iron, depleted body iron stores, ZPP, and ZPP/Hb ratio. Appropriate interventions are urgently needed to address the causes of IDA among pregnant teenagers.

Order of Authors:
Reginald Adjetey Annan, MPhil
Linda Afriyie Gyimah
Charles Apprey
Anthony Kwaku Edusei

Powered by Editorial Manager® and ProduXion Manager® from Aries Systems Corporation

	Odeafo Asamoah-Boakye
	Linda Nana Esi Aduku
	Wisdom Azanu
	Herman E. Lutterodt
**Additional Information:**	
**Question**	**Response**
**Financial Disclosure**<br><br>Enter a financial disclosure statement that describes the sources of funding for the work included in this submission. Review the submission guidelines for detailed requirements. View published research articles from *PLOS ONE* for specific examples.<br><br>This statement is required for submission and **will appear in the published article** if the submission is accepted. Please make sure it is accurate.<br><br>**Unfunded studies**<br>Enter: *The author(s) received no specific funding for this work.*<br><br>**Funded studies**<br>Enter a statement with the following details:<br>• Initials of the authors who received each award<br>• Grant numbers awarded to each author<br>• The full name of each funder<br>• URL of each funder website<br>• Did the sponsors or funders play any role in the study design, data collection and analysis, decision to publish, or preparation of the manuscript?<br>• **NO** - Include this sentence at the end of your statement: *The funders had no role in study design, data collection and analysis, decision to publish, or preparation of the manuscript.*<br>• **YES** - Specify the role(s) played.<br><br>* typeset	Funding for this study was provided by the Nestle Foundation. The funders had no role in study design, data collection and analysis, decision to publish, or preparation of the manuscript.
**Competing Interests**<br><br>Use the instructions below to enter a competing interest statement for this submission. On behalf of all authors, disclose any competing interests that	The authors have declared that no competing interests existed regarding the publication of this study.

could be perceived to bias this work—acknowledging all financial support and any other relevant financial or non-financial competing interests.<br><br>This statement **will appear in the published article** if the submission is accepted. Please make sure it is accurate. View published research articles from *PLOS ONE* for specific examples.<br><br>**NO authors have competing interests**<br><br>Enter: *The authors have declared that no competing interests exist.*<br><br>**Authors with competing interests**<br><br>Enter competing interest details beginning with this statement:<br><br>*I have read the journal's policy and the authors of this manuscript have the following competing interests: [insert competing interests here]*<br><br>* typeset	
**Ethics Statement**<br><br>Enter an ethics statement for this submission. This statement is required if the study involved:<br><br>• Human participants<br>• Human specimens or tissue<br>• Vertebrate animals or cephalopods<br>• Vertebrate embryos or tissues<br>• Field research<br><br>Write "N/A" if the submission does not require an ethics statement.<br><br>General guidance is provided below. Consult the submission guidelines for detailed instructions. **Make sure that all information entered here is included in the Methods section of the manuscript.**	Ethical approval for the study (Reference: CHPRE/ AP/236/18) was obtained from Committee on Human Research, Publications, and Ethics of the Kwame Nkrumah University of Science and Technology, Kumasi, Ghana (CHRPE/KNUST) and written personal or parental consent was obtained from participants before the study.

**Format for specific study types**<br><br>**Human Subject Research (involving human participants and/or tissue)**<br>• Give the name of the institutional review board or ethics committee that approved the study<br>• Include the approval number and/or a statement indicating approval of this research<br>• Indicate the form of consent obtained (written/oral) or the reason that consent was not obtained (e.g. the data were analyzed anonymously)<br><br>**Animal Research (involving vertebrate animals, embryos or tissues)**<br>• Provide the name of the Institutional Animal Care and Use Committee (IACUC) or other relevant ethics board that reviewed the study protocol, and indicate whether they approved this research or granted a formal waiver of ethical approval<br>• Include an approval number if one was obtained<br>• If the study involved *non-human primates*, add *additional details* about animal welfare and steps taken to ameliorate suffering<br>• If anesthesia, euthanasia, or any kind of animal sacrifice is part of the study, include briefly which substances and/or methods were applied<br><br>**Field Research**<br><br>Include the following details if this study involves the collection of plant, animal, or other materials from a natural setting:<br>• Field permit number<br>• Name of the institution or relevant body that granted permission	
**Data Availability**<br><br>Authors are required to make all data underlying the findings described fully available, without restriction, and from the time of publication. PLOS allows rare exceptions to address legal and ethical concerns. See the PLOS Data Policy and FAQ for detailed information.	No - some restrictions will apply

A Data Availability Statement describing where the data can be found is required at submission. Your answers to this question constitute the Data Availability Statement and **will be published in the article**, if accepted.<br><br>**Important:** Stating 'data available on request from the author' is not sufficient. If your data are only available upon request, select 'No' for the first question and explain your exceptional situation in the text box.<br><br>Do the authors confirm that all data underlying the findings described in their manuscript are fully available without restriction?	
**Describe where the data may be found in full sentences. If you are copying our sample text, replace any instances of XXX with the appropriate details.**<br><br>• If the data are **held or will be held in a public repository**, include URLs, accession numbers or DOIs. If this information will only be available after acceptance, indicate this by ticking the box below. For example: *All XXX files are available from the XXX database (accession number(s) XXX, XXX.)*.<br>• If the data are all contained **within the manuscript and/or Supporting Information files**, enter the following: *All relevant data are within the manuscript and its Supporting Information files.*<br>• If neither of these applies but you are able to provide **details of access elsewhere**, with or without limitations, please do so. For example:<br><br>*Data cannot be shared publicly because of [XXX]. Data are available from the XXX Institutional Data Access / Ethics Committee (contact via XXX) for researchers who meet the criteria for access to confidential data.*<br><br>*The data underlying the results presented in the study are available from (include the name of the third party*	Data cannot be shared publicly because it contains sensitive identifying information. However, data are available from the Committee on Human Research, Publication, and Ethics (CHRPE), the ethics board of the School of Medical Sciences of the Kwame Nkrumah University of Science and Technology, KNUST and Komfo Anokye Teaching Hospital (KATH), Kumasi, Ghana (Email: chrpe.knust.kath@gmail.com) for researchers who meet the criteria for access to confidential data.

and contact information or URL).<br>• This text is appropriate if the data are owned by a third party and authors do not have permission to share the data.<br><br>* typeset	
Additional data availability information:	

Click here to access/download;Manuscript;IDA associated factors in pregnant teenagers.docx

Factors associated with Iron Deficiency Anaemia among pregnant teenagers in Ghan[illegible]

Reginald Adjetey Annan[1*], Linda Afriyie Gyimah[1¶], Charles Apprey[1¶], Anthony Kwaku Edusei[4¶], Odeafo Asamoah-Boakye[1¶], Linda Nana Esi Aduku[1&], Wisdom Azanu[2&], Herman E. Lutterodt[3&]

[1]Department of Biochemistry and Biotechnology, Faculty of Biosciences, College of Science, Kwame Nkrumah University of Science and Technology, Kumasi, Ghana.

[2]Department of Obstetrics and Gynaecology, University of Allied Health Sciences, Ho, Ghana.

[3]Department of Food Science and Technology, Kwame Nkrumah University of Science and Technology, Kumasi, Ghana,

[4]Department of Community Health, School of Public Health, Kwame Nkrumah University of Science and Technology, Kumasi, Ghana.

* Corresponding author

Email: reggie@imtf.org

Abstract

Background

Iron Deficiency Anaemia (IDA) is reportedly high in pregnant adults and the causes well studied. However, among pregnant teenagers, the levels and associated factors of IDA are not fully understood.

Methods

In a prospective cohort study among Ghanaian pregnant teenagers, aged 13-19 years, IDA prevalence and associated factors were investigated. Sociodemographic data, household hunger scale, lived poverty index, eating behaviour, and interventions received during antenatal care (ANC) were obtained from 416 pregnant adolescents in Ashanti Region, Ghana. Anthropometric and nutrients intake using a repeated 24-hour dietary recall were determined and blood samples analysed for haemoglobin (Hb), serum levels

of ferritin, prealbumin, vitamin A, total antioxidant capacity (TAC), C-reactive protein (CRP), and zinc protoporphyrin (ZPP).

Results

Anaemia (Hb cutoff <11.0 g/dL) was 57.1%, deficient systemic supply of iron stores (31.4%), depleted body stores of iron (4.4%), inadequate dietary iron intake (97.6%), and inadequate multiple micronutrient intakes (52.2%) were notable among study participants. Between-subject effects using Generalized Linear Modelling indicated malaria tablet given at ANC (p=0.035), MUAC (p=0.043), ZPP (p<0.001), ZPP/Hb ratio (p<0.001) and depleted body iron stores (DBIS) (p<0.001) to significantly affect Hb levels. Pregnant teenagers with a high ZPP/Hb ratio (OR= 9.7, p<0.001, 95%CI= 6.0-15.8) had an increased odds of being anaemic compared to those with normal ZPP/Hb ratio. Participants who were wasted (OR= 1.2, p = 0.543, 95%CI= 0.6-2.3), those with depleted iron stores (OR= 3.0, p = 0.167, 95%CI= 0.6-14.6) had an increased odds of being anaemic. Participants who experienced hunger (OR= 2.9, p = 0.040, 95%CI= 1.1-7.8) had an increased odd of depleted iron stores compared to those who did not experience hunger. Participants with inadequate multiple micronutrients intakes (OR= 2.6, p = 0.102, 95%CI= 0.8-8.4), and those with low serum levels of ferritin (OR= 3.3, p = 0.291, 95%CI= 0.4-29.2) had increased odds of depleted body iron stores.

Conclusions

IDA is common among pregnant teenagers and the related factors include malaria tablets given at ANC, maternal hunger, maternal MUAC, deficient systemic supply of iron, depleted body iron stores, ZPP, and ZPP/Hb ratio. Appropriate interventions are urgently needed to address the causes of IDA among pregnant teenagers.

Keywords

Iron deficiency anaemia, pregnant teenagers, associated ris[illegible]

Introduction

Adolescence, a critical period of physical growth, early learning, and mental developmental changes have gained public interest, and is being seen as a life state suited for strategic health policies and commitments [1, 2]. Ghana has a youthful population, with the adolescent age group comprising about 22.0% of the total population [3]. Of all the health problems faced by adolescents, teenage pregnancy is arguable the most devastating due to its negative consequences on the health of the mother and infant [2]. Globally, it is estimated that 12 million girls aged 15-19 years have a child annually, and at least 770,000 of these childbirth takes place in developing countries [4]. The current Ghana demographic health survey shows that 14% of girls aged between 15 and 19 years had begun childbearing, with 11% live birth rate [5]. It is also known that early pregnancy and postnatal complications such as unsafe abortion, stillbirth, and pregnancy-induced hypertension are the main causes of mortality among girls aged 15-19 years old worldwide [6-8]. Iron deficiency anaemia is also a common complication that increases the risk of adverse pregnancy outcomes, such as low birth weight, preterm birth, perinatal and infant mortality, postpartum haemorrhage, and spontaneous abortion [9,10]. This implies that pregnant adolescent girls face a double burden of health risk because they have increased nutrients demand to ensure their own growth/development, in addition to that of their growing fetuses. Consequently, the UN's Sustainable Development Agenda on reducing poverty and hunger, promoting healthy lives and wellbeing at all ages, empowering women, and achieving equal education may not be attained if certain capacities of the adolescents are not developed [11].

Maternal anaemia has been a long-standing interest in the public health domain [12]. Among all the pathological causes of anaemia in pregnancy, iron deficiency anaemia is the most frequent, particularly in low-and-middle-income countries (LMICs), where the contribution from other anaemia disorders such as malaria and sickle cell disease are less significant [12, 13, 14]. Maternal anaemia occurs when haemoglobin concentration is less than 11.0g/dL, while in the absence of inflammation, ferritin

concentration less than 15 ug/L is also used to determine iron-deficiency anaemia [15]. According to WHO, more than 40 percent of maternal women globally are anaemic due to iron deficiency [16], and/or folate and other micronutrient deficiencies [17,18]. Among the many health interventions including antenatal and postnatal maternal care services, the Community-based Health Planning and Services initiatives in poorly resourced communities, and iron-folic acid supplementation given to pregnant women to reduce the burden of anaemia [19], Ghana is still reporting a high prevalence of anaemia (45.1%) among pregnant women aged 15-49 [20]. A recent retrospective study by Ampiah et al. [21] also reported a very high prevalence of anaemia among adults and teenage pregnant women, higher among teenagers, and not much reduced over five years.

There are two mains forms of iron deficiency; absolute and functional. Absolute iron deficiency occurs as a result of low or depleted body stores of iron while, functional iron deficiency is a disorder that occurs when total body stores are normal or increased, but the iron supply to the bone marrow is deficient [22, 23]. The two forms can coexist in pregnancy when risk factors are present [23]. The known causes of iron deficiency anaemia in pregnant women have been poor dietary intake as a result of food deprivation, consumption of less diverse foods, leading to poor nutritional status, and low iron stores, and therefore anaemia [24-27]. During pregnancy, there is an additional requirement for iron and folic acid to meet the pregnant woman's own nutritional needs as well as those of the developing foetus [16]. This is more so for the adolescent, who require nutrients for growth and development spurt experienced during this age as well. The reasons for the perpetual high anaemia prevalence are not obvious, but possibly, the underlying factors have not been fully investigated or understood. Researchers on anaemia has focused on adult pregnant women, and research interest into risk factors focused on related-causes such as; intestinal parasitic infections [28], malaria, HIV infections [29, 30], sickle cell anaemia [31]. Other studies in Ghana have shown that multiparity, maternal underweight [32], poor knowledge about anaemia [33],

malaria infection, no fish/meat consumption during pregnancy [34] are all predicting factors of anaemia among pregnant women that affect pregnancy outcomes.

Evidence from the above literature concludes that maternal anaemia has multifactorial causes, that negatively affect maternal and birth outcomes, but these are under study among pregnant teenagers. The interactive effects of both immediate and underlying causes of anaemia: socio-demographic, antenatal intervention uptake, poverty and hunger, dietary factors, anthropometric and nutritional status on anaemia among Ghanaian pregnant teenagers are not known. Our study sought to investigate these factors to deepen our understanding of the determinants of anaemia during pregnancy.

Methods

Study setting

Teenage pregnant girls were recruited from 29 communities in Kumasi Metropolis, Asante Akim Central, Ejisu Juaben, Bosomtwi, Asante Akim South and North and Ahafo Ano North and South Districts, all in Ashanti Region, Ghana, from May to August 2018. Ashanti Region has an estimated population of 5,792,187, accounting for 19% of Ghana's total population in 2019 [35]. The report from the Ghana Maternal Health Survey 2017 showed a high prevalence of teenage pregnancy (12.2%) and anaemia in the region [36]. The study areas had hospitals, health centres, or Community-based Health Planning Services (CHPS) compounds [5]. The study population consisted of pregnant teenagers (aged 13 to 19 years old) with gestational age up to 32 gestational weeks, who were attending antenatal clinics at their district health facilities.

Study Design

The study is an excerpt from a hospital-based prospective cohort study, involving 416 pregnant teenagers, aged 13 to 19 years old, residing in the 29 communities. The main study recruited and collected baseline

data on the pregnant teenagers. They were subsequently followed and birth outcomes evaluated upon delivery. The current study focuses on the baseline characteristics of the participants.

Sample size and sampling

A sample size of 420 was calculated following a previous report by Ayensu et al. [37], that reported a low birth weight prevalence of 23% and a marginal error of 5%. A convenience sampling method using a first come first served approach was used in recruiting participants from hospitals, health centres, and CHPS zones during antenatal care visits. We obtained days in which antenatal clinics were held for pregnant adolescents in the hospitals/health centres involved in this study. On these dates, research assistants visited the hospitals/health centres, and any pregnant teenager within the required age group (13-19 years) was eligible for recruitment. Although special antenatal clinics were held for teenagers, attendance was low due to stigma. Hence, in the rural districts, community information centres were asked to announce and invite all pregnant adolescents to the health centres. Those that came and consented to the study were recruited.

Ethics

The study was approved by the Council for Scientific and Industrial Research (CSIR), Ghana (Reference: CHPRE/ AP/ 236/18). Study protocols/aims were first explained to all participants in their local language (Asante Twi). Written and signed informed consent was obtained from all participants by following CSIR protocols for recruiting for the study.

Data Collection

Data were collected on socio-demographic variables, dietary diversity, eating behavious, availability of food and poverty, and dietary intake. Anthropometric measurements were done and blood samples collected for haematology and biochemical analysis. Antenatal interventions uptake, and other pregnancy-related practices were collected with a structured questionnaire. Data on age and parity were

verified from their National Health Insurance Identification cards and maternal health record books of participants.

Dietary Assessment and Eating Behaviour

A repeated 24-hour dietary recall on 2 weekdays and 1 weekend was used to obtain dietary intake of the participants to determine nutrients intake. Household food measures were used to quantify food consumed by pregnant teenagers, and these were later converted into grams using a standardized excel spreadsheet which has the measuring weight of Ghanaian foods. These grams were further computed into a nutrient analysis Microsoft Excel software designed by the University of Ghana, Department of Food Science and Nutrition [38] which included mostly consumed Ghanaian foods. Average nutrients intakes of macro and micronutrients were calculated by the software, and mean nutrients intakes were compared with recommended dietary allowances (RDAs)/estimated average requirement (EAR) [39] to establish adequacies and inadequacies of nutrient intakes. Besides, we also determined participants who had combined adequacies/inadequacies for iron, folate, and vitamin B_{12} when compared with the RDA, and this was reported as multiple micronutrient intake (MMI). Participants who had three inadequate intakes for iron, folate, and vitamin B_{12} were termed as inadequate multiple micronutrient intake (IMMI), inadequate for both iron-folate was classified as inadequate iron-folate intake, and inadequate for both iron-vitamin B_{12} was termed as inadequate iron-vitamin B_{12} intake. The same was done for combined adequacies/inadequacies for energy, carbohydrate, and protein, and reported as combined macronutrient intakes (CMI). Participants who had three inadequate intakes for energy, carbohydrate, and protein were termed inadequate macronutrient intakes (IMI).

Dietary diversity was assessed using a repeated 24-hour dietary recall method, taken for three days (two weekdays and one weekend). FAO's Women's Dietary Diversity Score (WDDS) was then used to assess the dietary diversity of participants [40]. The WDDS consisted of ten (10) food groups, namely; grains, white roots and tubers, plantain; pulses (beans, pea, lentils); nuts and seeds; dairy; meat, poultry, and fish; eggs;

dark green leafy vegetables; other vitamin A rich fruits and vegetables; other vegetables; other fruits [40]. We used the first 24-hour recall from each participant to score her dietary diversity using the 10 food groups. The WDDS was then classified as low (≤ 3), medium (4 – 5 food groups), or high (≥ 6 food groups) according to FAO's guidelines [41]. The WDDS was further regrouped into adequate (5 – 9) and inadequate (0 – 4) dietary diversity scores to help with statistical association for the population. A questionnaire was used to obtain data on food cravings, pica practices, and food aversions.

Household Hunger and Poverty status Assessment

The Household Hunger Scale (HHS) designed by Ballard [42], was adopted to assess food availability and deprivation of pregnant teenagers while the Lived Poverty Index (LPI) designed by Mattes [43] determining poverty status. The HHS assesses questions related to food availability and deprivation over the past month. The responses to these questions are coded and scored, ranging from 0 – 6. Scores less than 2 shows little or no hunger, 2 – 3 shows moderate hunger, and 4 – 6 shows severe hunger [42]. The Lived Poverty Index (LPI) assessed questions related to the availability of food, water, cash income, medical care, and cooking fuel over the past year. The responses are scored on a five-point scale, ranging from 0 (which can be thought of as no lived poverty) to 4 which reflects a constant absence of all primary necessities [43]. The averages of the scores were classified as low (0 – 0.5), low-moderate (0.51-1.0), high-moderate (1.1-1.5) and high (> 1.5) LPI.

Assessment of Anthropometric Status

Weight, height and mid-upper arm circumference (MUAC) of participants were determined. A weighing scale (model: DT602, India) was used to measure the weight of participants, to the nearest 0.1kg while, height measurement in centimeter was taken using a portable stadiometer (Secca 213, India), all without shoes on. The values obtained for weight and height were used to calculate the body mass index (BMI) of the participants and categorized using the WHO 2004 classification for BMI [44]. MUAC measurement was

determined as a proxy indicator of maternal weight status since it has good specificity in determining weight during pregnancy [45, 46]. An inelastic tape measure was used to take MUAC of participants by locating the midpoint between the acromion and olecranon bone on the left hand of participants. MUAC measurement less than 22.0cm was referred to as severe wasting, 22.0cm to less than 24.0cm as mild/moderate wasting, and 24.0cm and above as normal MUAC [47]. All anthropometric measurements were done in duplicates and averages were used.

Determination of haemoglobin (Hb) levels

Two milliliters venous blood sample of participants collected into anticoagulant EDTA tubes was used to determine haemoglobin levels of participants using Sysmex Haematology system (USA). The WHO cut-off for Hb was used to determine anaemia prevalence among the participants; haemoglobin values less than 11.0g/dL means anaemia, 11.0g/dL and above means no anaemia [15].

Assessment of serum nutrients

Serum ferritin is recommended by WHO in the assessment of iron levels, in the absence of inflammation [48]. However, serum ferritin is likely to be high in the presence of inflammation or infection [49]. Thus, an inflammatory marker, CRP, was also assessed. Five milliliters venous blood taken from participants into serum gel separator tubes were centrifuged at 4000 rpm for 5 minutes to obtain the serum. The serum was used for the analysis of serum ferritin, prealbumin, c-reactive protein (CRP), total antioxidant capacity (TAC) and zinc protoporphyrin (ZPPP) using a sandwich enzyme-linked immunosorbent assay (ELISA) techniques (from R&D system Inc, USA) at the Clinical Analysis Laboratory of the Department of Biochemistry and Biotechnology, Kwame Nkrumah University of Science and Technology. The optical density for serum ferritin, serum prealbumin, and serum ZPP was taken at 450 nm wavelength within 15 minutes, using a multipurpose microplate ELISA reader (Mindray MR-96A). TAC was determined using the ferric reducing ability of plasma (FRAP) assay protocol described by Benzie and Strain [50]. TAC was

measured at wavelength 593nm, with the help of a spectrophotometer (Mindray BA-88A, China). The standard curves of known concentrations of the respective recombinant value were used for the calculation of biochemical variables. All biochemical analyses were done in duplicates. Serum iron deficiency was termed as serum ferritin less than 15 µg/L [51]. Low serum prealbumin was defined as serum levels less than 50-500 mg/L [52]. ZPP values were measured as µg/dL and converted to µmol/mol heme (ZPP/Hb ratio) by calculating the following formula used by Stanton et al. [53] and Yu [54]. ZPP/Hb ratio = $\frac{\mu\text{mol of ZPP}}{\text{mol of haemoglobin}} = \frac{\mu\text{g of ZPP}}{\text{gram of Hb}} \times 25.8$

Deficient systemic iron supply was defined as ZPP/Hb ratio greater than 80 µmol/mol heme, ZPP/Hb ratio between 60-80 µmol/mol heme was termed moderate-high, and less than 60 µmol/mol heme means adequate systemic iron supply [55]. Depleted body iron stores (DBIS) were defined as participants with ZPP/Hb ratio greater than 80 µmol/mol heme, and serum ferritin less than 20µg/L.

Data analysis

All data were analysed using IBM Statistical Package for Social Sciences version 25 (SPSS IBM Inc Chicago, USA). Absolute and relative frequencies were determined for sociodemography, hunger status, poverty status, eating behaviour, dietary diversity, antenatal intervention uptake, nutrient intakes, anthropometric, haemoglobin level, and biochemical variables. Kolmogorov-Smirnov test of normality was performed to determine whether all continuous variables met parametric assumptions. A chi-square (Fisher’s exact test) cross-tabulation was performed to compare frequencies of hunger status, poverty status, eating behaviour, women’s dietary diversity score, antenatal intervention uptake, nutrient intakes, anthropometric, haemoglobin level, biochemical variables, and iron deficiency anaemia status. Presenting data as mean±SD for the continuous variable, the mean differences of household hunger scale, lived poverty index, women’s dietary diversity score, anthropometric and biochemical variables were compared by iron deficiency anaemia status. An independent t-test and a two-way analysis of variance

(ANOVA) (Generalized Linear Model test) were used for parametric comparisons, while Mann Whitney 'U' test was performed for non-parametric comparisons of all continuous variables. Univariate tests of variables associations were performed using the generalized linear model test to determine the combined effect of study variables on haemoglobin level. Study variables that were significant at Chi-square/ANOVA analysis were put into the Binary logistic regression model to determine predictors of iron deficiency anaemia. All tests were 2-tailed, and p-values < 0.05 were termed significant.

Results

Figure 1 indicates the prevalence of iron-deficiency anaemia among pregnant teenagers. Anaemia prevalence by haemoglobin status was 57.1%, and 31.4% of the participants had poor systemic supply of body iron stores during pregnancy. Additionally, 4.4% of the pregnant teenagers were depleted of body stores of iron. In terms of dietary iron deficiency anaemia, 97.6%, of the participants had inadequacy for iron, 90.4%, 54.6% of them had combined inadequacies for iron-folate, and iron-vitamin B_{12} intakes respectively. Also, 52.2% of the pregnant adolescent had inadequate multiple micronutrient intakes (iron-folate-vitamin B_{12} deficiencies) when compared with RDA.

Fig 1. Iron-deficiency anaemia prevalence among pregnant teenagers

Supplement 1 shows the relationship between sociodemographic characteristics and anaemia prevalence. Anaemia prevalence did not significantly vary by sociodemographic factors (p>0.05). However, more rural (60.9%) than urban (54.4%) pregnant teenagers were anaemic (p = 0.222), older pregnant teenagers (16-19 years) were more anaemic than their younger (13-15 years) counterparts (57.6% versus 51.6%, p = 0.573), and the unmarried (59.4%) were more anaemic than the married (50.0%, p = 0.103). Moreover, anaemia prevalence was lower among participants who had more than one pregnancy (51.0%) than those with their first pregnancy (59.1%, p = 0.162). Participants who had

completed SHS school (61.0%), and those with income between ₵500- ₵1000 (75.0%) presented the highest prevalence of anaemia.

Supplement 1. Relationship between sociodemographic and haemoglobin status

		Hb status			
**Variables**	**Total**	**Anaemia**	**No anaemia**	**Chi-square**	**P value**
**Community type**					
Rural	173(41.6)	103(60.9)	66(39.1)	1.711	0.222‡
Urban	243(58.4)	129(54.4)	108(45.6)		
**Age group (years)**					
13-15	32 (7.7)	16(51.6)	15(48.4)	0.419	0.573‡
16-19	384 (92.3)	216(57.6)	159(42.4)		
**Marital status**					
Single	316 (76.0)	183(59.4)	125(40.6)	2.691	0.103‡
Married	100 (24.0)	49(50.0)	49(50.0)		
**Educational level**					
None	19 (4.6)	10(52.6)	9(47.4)	0.750	0.861¥
Primary	56 (13.5)	31(55.4)	25(44.6)		
JHS	255 (61.3)	141(56.6)	108(43.4)		
SHS	86 (20.7)	50(61.0)	32(39.0)		
**Occupation status**					
Unemployed	298 (71.6)	162(55.7)	129(44.3)	0.910	0.374‡
Employed	118 (28.4)	70(60.9)	45(39.1)		
**Parity**					
One	316 (76.0)	182(59.1)	126(40.9)	1.977	0.162‡
More than one	100 (24.0)	50(51.0)	48(49.0)		
**Income (₵)**					
None	310 (74.5)	169(56.1)	132(43.9)	1.307	0.727‡
Below 100	41 (9.9)	26(63.4)	15(36.6)		
Between 100- <500	61 (14.7)	34(56.7)	26(43.3)		
Between 500-1000	4 (1.0)	3(75.0)	1(25.0)		

Data are presented as frequency (percentage)¥- Chi-square P value, ‡- Fisher's exact P value

Table 1 presents the relationship between iron deficiency status, and household hunger scale, lived poverty, eating behaviours, and intervention received at ANC. Over 8 in 10 pregnant teenagers with depleted body iron stores had inadequate women's dietary diversity score (WDDS) compared with less than 6 in 10 (57.4%, p = 0.046) among those with normal body iron stores. Also, about 30% of pregnant teenagers who were depleted of body iron stores suffered severe hunger compared with 11% of those

with normal body iron stores (p = 0.029). However, the proportion of participants who had depleted body iron stores and those with normal body iron stores did not significantly vary by poverty status (p = 0.633), food aversion (p = 0.133), food craving (p = 0.797), and whether the following services were received during ANC or not: nutrition education (p = 0.808), micronutrient supplementation (p = 0.625), and malaria tablet given (p = 0.315).

The proportion of participants who were anaemic (low Hb) and non-anaemic did not significantly differ by household hunger status (p= 0.310), poverty status (p = 0.318), food aversion (p = 0.615), food craving (p = 0.531), pica practice (p = 0.304), women's dietary diversity score (p = 0.359), nutrition education received (p = 0.617), micronutrient supplement (p = 0.617), and malaria tablet given (p = 0.610) at ANC. However, severe hunger among anaemic girls was higher (13.4%) than among the non anaemic participants (9.2%), (p = 0.310), and poverty was higher in anaemic girls (37.5%) than the non anaemic participants (31.6%), p = 0.318, and inadequate WDDS in anaemic girls (61.2%) was higher than in non anaemic participants (56.3%), p = 0.359.

Table 1. Iron deficiency status, and household hunger scale, lived poverty index, eating behaviours and intervention received at ANC

		Hb status			Body iron stores		
**Variables**	**Total**	**Anaemia**	**No anaemia**	**X^2, (P value)**	**Depleted**	**Normal**	**X^2, (P value)**
**HHS**							
No Hunger	303 (72.8)	164(70.7)	134(77.0)	2.34(0.310)[ǂ]	8(47.1)	276(74.2)	7.10(**0.029**)[ǂ]
Moderate Hunger	63 (15.1)	37(15.9)	24(13.8)		4(23.5)	55(14.8)	
Severe Hunger	50 (12.0)	31(13.4)	16(9.2)		5(29.4)	41(11.0)	
**Mean HHS**	0.9±0.1	1.0±0.1	0.8±0.1	0.389	1.8±0.5	0.9±0.1	**0.043**
**Lived Poverty Index(LPI)**							
Low Moderate	194 (45.2)	107(46.1)	82(47.1)	2.29(0.318)[¥]	6(35.3)	174(46.8)	0.91(0.633)[ǂ]
High Moderate	75 (18.3)	38(16.4)	37(21.3)		4(23.5)	66(17.7)	
High	152 (36.5)	87(37.5)	55(31.6)		7(41.2)	132(35.5)	
**Mean LPI**	1.5±0.0	1.5±0.0	1.4±0.0	0.414	1.6±0.1	1.5±0.0	0.365
**Food Aversion**							
Yes	185 (44.5)	106(45.7)	75(43.1)	0.26(0.615)[ǂ]	11(64.7)	164(44.1)	2.79 (0.133)
No	231 (55.5)	126(54.3)	99(56.9)		6(35.3)	208(55.9)	
**Food Craving**							
Yes	267 (64.2)	152(65.5)	108(62.1)	0.51(0.531)[ǂ]	12(70.6)	238(64.0)	0.30(0.797)[ǂ]
No	149 (35.8)	80(34.5)	66(37.9)		5(29.4)	134(36.0)	
**Pica Practice**							
Yes	160 (38.5)	95(40.9)	62(35.6)	1.18(0.304)[ǂ]	6(35.3)	145(39.0)	0.09(1.000)[ǂ]
No	256 (61.5)	137(59.1)	112(64.4)		11(64.7)	227(61.0)	
**Women's DDS (3 categories)**							
Low	108 (26.0)	67(28.9)	36(20.7)	3.55(0.169)[¥]	4(23.5)	93(25.0)	0.19 (0.908)
Medium	285 (68.5)	134(57.8)	111(63.8)		11(64.7)	223(59.9)	
High	23 (5.5)	31(13.4)	27(15.5)		2(11.8)	56(15.1)	
**WDDS (2 categories)**							
Inadequate	247 (59.4)	142(61.2)	98(56.3)	0.98(0.359)[ǂ]	14(82.4)	214(57.5)	4.13(**0.046**)[ǂ]
Adequate	169 (40.6)	90(38.8)	76(43.7)		3(17.6)	158(42.5)	
**Mean WDDS**	4.3±0.1	4.2±0.1	4.4±0.1	0.121	3.9±0.3	4.3±0.1	0.193
**Take MS**							
Yes	216(51.9)	118(50.9)	93(53.4)	0.26(0.617)[ǂ]	10(58.8)	191(51.3)	0.36(0.625)[ǂ]
No	200(48.1)	114(49.1)	81(46.6)		7(41.2)	181(48.7)	
**NE received**							
Yes	206(49.5)	118(50.9)	84(48.3)	0.26(0.617)[ǂ]	9(52.9)	182(48.9)	0.10(0.808)[ǂ]
No	210(50.5)	114(49.1)	90(51.7)		8(47.1)	190(51.1)	
**Take Malaria Tablet**							
Yes	168(40.4)	97(41.8)	68(39.1)	0.30(0.610)[ǂ]	9(52.9)	147(39.5)	1.22(0.315)[ǂ]
No	248(59.6)	135(58.2)	106(60.9)		8(47.1)	225(60.5)	

□Data are presented as frequency (percentage), □- Mean±SEM (standard error mean), □- Mann Whitney test performed, ¥- Chi-square P value, ǂ- Fisher's exact P value, Hb- Haemoglobin, HHS- Household Hunger Scale, WDDS- Women's Dietary Diversity Score, MS- Micronutrient Supplement, NE- Nutrition Education, bold values are significant at p<0.05.

Supplement 2 indicates the relationship between the proportions of nutrient intake adequacies and inadequacies by iron deficiency anaemia. The proportion of participants who had depleted body iron stores and those normal body iron stores did not significantly vary by nutrients intake (p>0.05), except for multiple micronutrient intake (iron-folate-vitamin B_{12}) whereby more pregnant teenagers with depleted of body iron stores (82.4%) had inadequate multiple micronutrient intakes than those with normal body iron stores (57.4%, p = 0.046). Moreover, participants with inadequate vitamin B_{12} (76.5% versus 52.7%), and inadequate iron-vitamin B_{12} (100% versus 89.1%) intakes were markedly higher among those with depleted body iron stores compared with normal body iron stores.

Supplement 2. Relationship between nutrient intake and iron deficiency anaemia

		Hb status			Body iron stores		
**Variables**	**Total**	**Anaemia**	**No anaemia**	**x^2 (P value)**	**Depleted**	**Normal**	**x^2 (P value)**
**Energy**							
Inadequate	350(84.1)	196(84.5)	145(83.3)	0.09 (0.785)	14(82.4)	312(83.9)	0.02(0.745)
Adequate	66(15.9)	36(15.5)	29(16.7)		3(17.6)	60(16.1)	
**Carbohydrate**							
Inadequate	70(16.8)	43(18.5)	25(14.4)	1.23 (0.285)	4(23.5)	61(16.4)	0.59 (0.502)
Adequate	346(83.2)	189(81.5)	149(85.6)		13(76.5)	311(83.6)	
**Protein**							
Inadequate	321(77.2)	179(77.2)	135(77.6)	0.01 (1.000)	14(82.4)	285(76.6)	0.30 (0.772)
Adequate	95(22.8)	53(22.8)	39(22.4)		3(17.6)	87(23.4)	
**CMI**							
Inadequate	308(74.0)	168(72.4)	133(76.4)	0.83 (0.423)	13(76.5)	274(73.7)	0.06 (1.000)
Adequate	108(26.0)	64(27.6)	41(23.6)		4(23.5)	98(26.3)	
**Iron**							
Inadequate	406(97.6)	226(97.4)	170(97.7)	0.03 (1.000)	17(100.0)	363(97.6)	0.42 (1.000)
Adequate	10(2.4)	6(2.6)	4(2.3)		0(0.0)	9(2.4)	
**Folate**							
Inadequate	377(90.6)	212(91.4)	155(89.1)	0.60 (0.497)	17(100.0)	334(89.8)	1.92 (0.392)
Adequate	39(9.4)	20(8.6)	19(10.9)		0(0.0)	38(10.2)	
**Vitamin B_{12}**							
Inadequate	226(54.3)	123(53.0)	97(55.7)	0.29 (0.615)	13(76.5)	196(52.7)	3.69 (0.080)
Adequate	190(45.7)	109(47.0)	77(44.3)		4(23.5)	176(47.3)	
**Iron-Folate**							
Inadequate	376(90.4)	213(91.8)	153(87.9)	1.68 (0.239)	17(100.0)	333(89.5)	1.98 (0.396)
Adequate	40(9.6)	19(8.2)	21(12.1)		0(0.0)	39(10.5)	
**Iron-Vitamin B_{12}**							
Inadequate	227(54.6)	124(53.4)	97(55.7)	0.21 (0.687)	13(76.5)	197(53.0)	3.61 (0.080)
Adequate	189(45.4)	108(46.6)	77(44.3)		4(23.5)	175(47.0)	
**MMI (iron, folate, vitamin B_{12})**							
Inadequate	217(52.2)	119(51.3)	92(52.9)	0.09 (0.764)	13(76.5)	187(50.3)	4.46 (**0.046**)
Adequate	199(47.8)	113(48.7)	82(47.1)		4(23.5)	185(49.7)	

Data are presented as frequency (percentage), CMI- Combined Macronutrient Intake (energy, carbohydrate, and protein), MMI- Multiple Micronutrient Intake (Iron, folate, and vitamin B_{12}), Fisher's exact P value.

Table 2 presents the relationship between anthropometric status and anaemia (low Hb). The mean MUAC was higher among pregnant teenagers who were not anaemic (26.6±0.2cm) than those who were anaemic

(25.8±0.2cm, p = 0.006) respectively. The proportion of participants who were anaemic and non-anaemic did not significantly differ by BMI status (p = 0.417), and MUAC status (p = 0.319), although underweight (2.6%), and severe wasting (6.5%) were both higher among anaemic participants than non anaemic (underweight 1.1%, severe wasting 4.6%) participants.

The proportion of participants who had depleted body iron and those with normal body iron stores did not significantly vary by BMI status (p = 0.147), and MUAC status (p = 0.454).

Table 2. Relationship between anthropometric status and iron deficiency anaemia

		Hb status			Body iron stores		
**Variable**	**Total**	**Anaemia**	**No anaemia**	**X^2 (P value)**	**Depleted**	**Normal**	**X^2 (P value)**
**BMI status(Kg/m²)**□							
**mean BMI**⍰	23.1±0.1	22.9±0.2	23.5±0.2	0.106⍰	23.0±1.0	23.1±0.2	0.319⍰
Underweight	8(1.9)	6(2.6)	2(1.1)	2.8(0.417)‡	0(0.0)	2(2.2)	5.36(0.147)‡
Normal	294(70.7)	168(72.4)	118(67.8)		13(76.5)	261(70.2)	
Overweight	100(24.0)	50(21.6)	48(27.6)		2(11.8)	92(24.7)	
Obese	14(3.4)	8(3.4)	6(3.4)		2(11.8)	11(3.0)	
**MUAC status(cm)**□							
**Mean MUAC**⍰	26.2±0.1	25.8±0.2	26.6±0.2	**0.006**⍰	26.1±0.9	26.2±0.2	0.559⍰
Severe wasting	23(5.5)	15(6.5)	8(4.6)	2.28(0.319)¥	1(5.9)	22(5.9)	1.50(0.454)‡
Moderate/mild wasting	97(23.3)	58(25.0)	35(20.1)		6(35.3)	83(22.3)	
Normal	296(71.2)	159(68.5)	131(75.3)		10(58.8)	267(71.8)	

□Data are presented as frequency (percentage), ⍰- Mean±SEM (standard error mean), ⍰- Mann Whitney test, ¥- Chi-square P value, ‡- Fisher's exact P value, Hb- Haemoglobin,, BMI- Body Mass Index, MUAC- Mid-Upper Arm Circumference, bold value is significant at p < 0.05.

The relationship between serum nutrients status and iron deficiency anaemia (low Hb and depleted body iron stores) status is presented in Table 3. The proportion of participants with a high ZPP/Hb ratio, indicating poor systemic iron supply was higher in anaemic (49.3%) compared with non anaemic (7.7%) participants (p<0.001). The mean levels of serum ZPP (p<0.001), and ZPP/Hb ratio (p<0.001) were significantly higher among pregnant teenagers who were anaemic (38.3±2.9µmol/L, 109.6±9.0) than those who were not anaemic (28.4±2.0µmol/L, 64.9±4.6) respectively. The proportion of anaemic and

non-anaemic participants with low serum vitamin A (p = 0.373), serum ferritin (p = 0.785), serum prealbumin (p = 0.763) did not significantly differ. Also, the mean levels of serum c-reactive protein (p = 0.066) and total antioxidant capacity (p = 0.860) did not significantly differ between anaemic and non-anaemic participants. For serum iron status, more anaemic participants (6.8%) had depleted iron stores than those who were not anaemic (1.2%, p = 0.010).

Almost all participants with deficient systemic iron supply (high ZPP/Hb ratio) (94.1%) had depleted body iron stores compared with less than a third (28.5%) among those with normal body iron stores (p<0.001). The mean levels of serum ferritin (36.9±0.8μg/L versus 17.4±0.7μg/L p<0.001), and prealbumin (12.7±0.7mg/L vs 8.0±0.5mg/L, p = 0.007) were higher among pregnant teenagers with normal body iron stores than those who had depleted body iron stores, respectively. Moreover, participants with normal body iron stores had a lower mean levels of serum ZPP (33.9±2.0μmol/L vs 37.5±6.0μmol/L, p = 0.001), and ZPP/Hb ratio (89.4±5.8 vs 109.1±14.9, p<0.001), compared to those who had depleted body iron stores respectively.

Table 3. Relationship between biochemical parameters and iron deficiency anaemia status

Variable	Total	Hb status		P value	Body iron stores		P value
		Anaemia	No anaemia		Depleted	Normal	
**Mean serum vit A**⍰	0.7±0.1	0.8±0.1	0.7±0.1	0.106⍰	0.8±0.3	0.7±0.1	0.910⍰
**Serum vitamin A**□ **(mmol/L**)							
Low	336(86.4)	194(87.8)	142(84.5)	0.373ǂ	12(70.6)	324(87.1)	0.066ǂ
Normal	53(13.6)	27(12.2)	26(15.5)		5(29.4)	48(12.9)	
**Mean serum ferritin**⍰	36.0±0.8	36.7±1.2	35.1±1.2	0.394⍰	17.4±0.7	36.9±0.8	**<0.001**⍰
**Serum ferritin status**□ **(µg/L)**							
Low	14(3.6)	9(4.1)	5(3.0)	0.785ǂ	1(5.9)	13(3.5)	0.471ǂ
Normal	375(96.4)	212(95.9)	163(97.0)		16(94.1)	359(96.5)	
**Mean serum PAB**	12.4±0.7	12.3±0.9	12.6±1.0	0.445⍰	8.0±0.5	12.7±0.7	**0.007**⍰
**Serum PAB(mg/L)** □							
Low	382(97.2)	216(96.9)	165(97.6)	0.763ǂ	17(100.0)	361(97.0)	1.000ǂ
Normal	11(2.8)	7(3.1)	4(2.4)		0(0.0)	11(3.0)	
**Mean ZPP/Hb ratio**⍰	90.3±5.6	109.6±9.0	64.9±4.6	**<0.001**⍰	109.1±14.9	89.4±5.8	**<0.001**⍰
**ZPP/Hb ratio**)□							
Normal	150(38.6)	37(16.7)	113(67.3)	**<0.001**¥	1(5.9)	149(40.1)	**<0.001**ǂ
Moderately high	117(30.1)	75(33.9)	42(25.0)		0(0.0)	117(31.5)	
High	122(31.4)	109(49.3)	13(7.7)		16(94.1)	106(28.5)	
**Mean ZPP (µg/dL)**⍰	34.0±1.0	38.3±2.9	28.4±2.0	**<0.001**⍰	37.5±6.0	33.9±2.0	**0.001**⍰
**Mean CRP (mg/L)**⍰	5.0±0.4	5.0±0.6	5.0±0.6	0.066⍰	5.1±2.1	5.0±0.4	0.385⍰
**Mean serum TAC**⍰	1.2±0.0	1.2±0.1	1.2±0.1	0.860⍰	1.1±0.1	1.2±0.0	0.974⍰
**Body iron stores**							
Depleted	17(4.4)	15(6.8)	2(1.2)	**0.010ǂ**			
Normal	372(95.6)	206(93.2)	166(98.8)				

□Data are presented as frequency (percentage), ⍰- Mean±SEM (standard error mean), ⍰- Mann Whitney test, ¥- Chi-square P value, ǂ- Fisher's exact P value, Hb- Haemoglobin, PAB- Prealbumin, ZPP- Zinc protoporphyrin, CRP- C-reactive protein, TAC- Total antioxidant capacity, bold values are significant at p < 0.05. ZPP/Hb measured in µmol/mol heme

Table 4 indicates how dietary factors, hunger status are associated with nutritional status and anaemia prevalence. Among those with inadequate MMI and depleted body iron stores, 84.6% were anaemic compared with 54.5% anaemia in adequate MMI and normal body iron stores. Among adequate MMI but

depleted body iron stores, anaemia was 100% compared with 56.2% anaemia among inadequate MMI and normal body iron store participants. Anaemia in no hunger iron-depleted participants was 100%, moderate hunger iron-depleted stores participants 50% and severe hunger iron-depleted stores participants 100%, compared to lower levels in no hunger normal iron stores (53.6%), moderate hunger normal body iron stores (60%) and severe hunger normal body iron stores participants (61%), p=0.010. Likewise, anaemia was higher in inadequate WDDS depleted body iron stores participants (85.7%), than inadequate WDDS normal body iron stores participants (56.4%), and adequate WDDS depleted body iron stores participants (100%), compared with normal body iron stores (53.8%), p=0.010.

Moreover, anaemia was consistently higher among the different categories of ZPP/Hb ratio; high, moderate, and normal (high signifying poor systemic iron supply) depleted body iron stores participants than the normal body iron stores participants, but highest among high ZPP/Hb ratio and also depleted body iron stores participants. Last but not the least, anaemia was highest among low serum ferritin-depleted body iron stores participants (100%), followed by normal serum ferritin-depleted body iron stores (87.5%), then low serum ferritin-normal body iron stores (61.5%) and the least were among normal ferritin-normal body iron stores (55.2%) participants (p=0.010).

Table 4. Interplay between MMI, HHS, WDDS, ZPP/Hb ratio, serum ferritin, body iron stores, and Hb status

		Haemoglobin (Hb) status			
**MMI**	**Variable**	**Anaemia**	**No anaemia**	**Chi-square**	**P value**
Inadequate	**Body Iron stores**				
	Depleted	11(84.6%)	2(13.4%)	7.154	**0.010**
	Normal	102(54.5%)	85(45.5%)		
Adequate	**Body Iron stores**				
	Depleted	4(100)	0(0.0)		
	Normal	104(56.2)	81(53.8%)		
**HHS**	**Body Iron stores**				
No hunger	Depleted	8(100)	0(0.0)	7.154	**0.010**
	Normal	148(53.6)	128(46.4)		
Moderate	**Body Iron stores**				
	Depleted	2(50)	2(50)		
	Normal	33(60)	22(40)		
Severe	**Body Iron stores**				
	Depleted	5(100)	0(0.0)		
	Normal	25(61)	16(39)		
**WDDS**	**Body Iron stores**				
Inadequate	Depleted	12(85.7)	2(14.3)	7.154	**0.010**
	Normal	121(56.4)	93(43.6)		
Adequate	**Body Iron stores**				
	Depleted	3(100)	0(0.0)		
	Normal	85(53.8)	73(46.2)		
**ZPP/Hb ratio**	**Body Iron stores**			7.154	**0.010**
Normal	Depleted	0(0.0)	1(100)		
	Normal	37(24.8)	112(75.2)		
Moderate high	**Body Iron stores**				
	Depleted	0(0.0)	0(0.0)		
	Normal	75(64.1)	42(35.9)		
High	**Body Iron stores**				
	Depleted	15(93.8)	1(6.2)		
	Normal	94(88.7)	12(11.3)		
**Serum ferritin**	**Body Iron stores**				
Low	Depleted	1(100)	0(0.0)	7.154	0.010
	Normal	8(61.5)	5(38.5)		
Normal	**Body Iron stores**				
	Depleted	14(87.5)	2(11.5)		
	Normal	198(55.2)	161(44.8)		

Data are presented as frequency (percentage), Fisher's exact P value, Hb- Haemoglobin, MMI- Multiple Micronutrient Intake, HHS- Household Hunger Scale, WDDS- Women's Dietary Diversity Score, bold values are significant at p < 0.05.

The results of between-subject effects, conducted in the univariate Generalized Linear Model are presented in Table 5. Taking into account all the multiple underlying factors of anaemia in the tests of associations on haemoglobin (Hb) levels, it was malaria tablet given at antenatal clinic (p = 0.035), MUAC (p=0.043), ZPP (p<0.001), ZPP/Hb ratio (p<0.001) and body iron stores (p<0.001) that had a significant effect on the Hb levels. Some variables (e.gs. lived poverty index, serum levels of vitamin A, C-reactive protein, prealbumin) which were not significant were removed, to report a few items in the table.

Table 5. Tests of between-subject effects of multiple variables on haemoglobin levels

**Tests of Between-Subjects Effects**					
**Dependent Variable: Hb levels**					
**Source**	**Type III Sum of Squares**	**Df**	**Mean Square**	**F**	**Significance**
Intercept	47.54	1	47.54	44.645	<0.001
Community type	0.886	1	0.886	0.832	0.362
Age	0.559	1	0.559	0.525	0.469
WDDS	0.008	1	0.008	0.007	0.933
HHS	0.032	1	0.032	0.03	0.862
Malaria tablet given	4.788	1	4.788	4.497	**0.035**
Dietary iron	0.758	1	0.758	0.712	0.399
MMI	0.05	1	0.05	0.047	0.829
MUAC	4.392	1	4.392	4.125	**0.043**
BMI	0.03	1	0.03	0.028	0.867
Serum ferritin	0.422	1	0.422	0.396	0.529
ZPP	158.095	1	158.095	148.469	**<0.001**
ZPP/Hb ratio	188.605	1	188.605	177.122	**<0.001**
Body iron store	18.086	1	18.086	16.985	**<0.001**
Error	387.599	364	1.065		

General Linear Model test for univariate parameters, BMI- Body mass index, Hb- Haemoglobin, TMS- Took Micronutrient Supplement, CMI- Combined Macronutrient Intake (energy, carbohydrate, and protein), MMI- Multiple Micronutrient Intake (Iron, folate and vitamin B_{12}), ZPP- Zinc protoporphyrin, Bold values are significant at p<0.05.

The predictors of anaemia and depleted body iron stores of the pregnant teenagers are presented in Table 6. Pregnant teenagers who had a high ZPP/Hb ratio (signifying poor systemic iron supply) (OR= 9.7,

p<0.001, 95%CI= 6.0-15.8) had more than 9 times increased odds of being anaemic compared to those who had normal ZPP/Hb ratio. Pregnant teenagers who were wasted (OR= 1.2, p = 0.543, 95%CI= 0.6-2.3), those who had depleted iron stores (OR= 3.0, p = 0.167, 95%CI= 0.6-14.6) had an increased odds of being anaemic, compared to those with normal MUAC or normal iron stores respectively.

Also, pregnant teenagers who experienced hunger (OR= 2.9, p = 0.040, 95%CI= 1.1-7.8) had over 2 times increased odds of having depleted body iron stores, compared to those who did not experience hunger. Participants who had inadequate multiple micronutrients intakes (OR= 2.6, p = 0.102, 95%CI= 0.8-8.4), those with low serum levels of ferritin (OR= 3.3, p = 0.291, 95%CI= 0.4-29.2) had an increased odds of depleted body iron stores. Pregnant teenagers who had inadequate women's dietary diversity score (OR= 0.3, p = 0.087, 95%CI= 0.1-1.2) had a lower odds of depleted body iron stores.

Table 6. Predictors of anaemia and depleted body iron stores

	Anaemia			95% Confidence Interval	
**Variable**	**β**	**OR**	**Significance**	**Lower**	**Upper**
**MUAC**					
Wasted	0.201	1.2	0.543	0.6	2.3
Normal		1.0			
**ZPP/Hb ratio**					
High	2.277	9.7	**<0.001**	6.0	15.8
Normal		1.0			
**Body iron stores**					
Depleted	1.109	3.0	0.167	0.6	14.6
Normal		1.0			
	**Depleted body iron stores**			**95% Confidence Interval**	
**Variable**	**β**	**OR**	**Significance**	**Lower**	**Upper**
**Serum ferritin**					
Low	1.181	3.3	0.291	0.4	29.2
Normal		1.0			
**Serum Prealbumin**					
Low	-17.346	0.01	0.999		
Normal		1.0			
**MMI**					
Inadequate	0.967	2.6	0.102	0.8	8.4
Adequate		1.0			
**HHS**					
Hunger present	1.049	2.9	**0.040**	1.1	7.8
No hunger		1.0			
**WDDS**					
Inadequate	-1.136	0.3	0.087	0.1	1.2
Adequate		1.0			
ZPP	-0.004	1.0	0.493	0.9	1.0

β – Slope/regression co-efficient, OR- Odds ratio, Hb- Haemoglobin, combined moderate and severe wasting, moderate and severe hunger, and moderate-high and high ZPP/Hb ratio for statistical strength bold values are significant at $p < 0.05$.

Discussion

The key findings of the study were the high prevalence of iron deficiency anaemia among teenagers. Iron deficiency anaemia (IDA) was common among the pregnant teenagers, from both systemic serum iron,

and their diet, and this is worrying. Our finding on IDA among teenagers is higher than that of pregnant adults in other studies in Ghana [20, 56, 57]. The studies by Ayensu et al. [57], the Ghana Demographic Health Survey [56], and the Ghana Micronutrients Survey [20] have reported that anaemia prevalence among pregnant adults was 56.0%, 44.6%, and 42.0% respectively. This means that compared to the adults, teenagers are more likely to be anaemic during pregnancy due to their high nutritional needs and immature body tissue growth. Higher anaemia among teenagers will more likely expose them to a greater risk of poor pregnancy outcomes (e.g. low birth weight, small-for-gestation age, stillbirth, preterm birth) than pregnant adults [16]. Almost 6 in 10 pregnant teenagers (57.1%) using Hb levels were anaemic, close to a third (31.4%) had deficient systemic supply of body iron stores, 4.4% had depleted body stores of iron, and almost all participants (97.6%) had inadequate dietary iron intake. Also, combined dietary inadequacies for iron-folate (90.4%) and iron-vitamin B_{12} intakes (54.6%), and multiple micronutrient deficiencies (52.2%) were very high, ranging from 5 to 9 in 10 participants. Iron deficiency anaemia is a public health problem among pregnant teenagers in Ghana. Studies conducted in Palestine and Ghana have reported a high prevalence of anaemia (25.0%, 70.0%, 52.0%), and depleted body iron stores (52.0%) among pregnant teenagers/adults [21, 32, 58]. Anaemia in pregnant teenagers is attributed to the fact that they are still growing and additional requirement for iron and folic acid to meet their own nutritional needs as well as those of the developing foetus during gravidity [16] but the very low intakes of nutrients that are linked with anaemia are equally attributable from this study.

Several studies have also reported sociodemographic status to affect iron deficiency anaemia in pregnancy [9, 32, 59]. In this study, we found significant associations between sociodemographic parameters of participants and iron deficiency anaemia. However, by comparison, the levels of anaemia among rural participants, older teenagers (16-19 years), unmarried teenagers, and employed participants were higher anaemic. Our findings are consistent with other studies that found no significant association between monthly income level, level of education, household size, and anaemia prevalence [58, 60] but

also contrary to other studies that reported certain socio-demographic characteristics, such as being a housewife, having less education, and multiparity to be associated with anaemia among pregnant women [9, 32, 59].

Food deprivation is associated with low systemic iron stores, and consequently anaemia [25]. For the adolescent, pregnancy can be a complicated issue due to the rapid increased demands of nutrients, especially for iron and folic acid, for the mother's blood expansion, growth as well as the foetus. Food deprivation and poor access to food are linked with poor dietary intakes, and the risk of micronutrient deficiencies, which can consequently lead to depleted body stores of nutrients [24, 27, 61]. We found hunger to predict depleted iron stores, with teenagers who suffered hunger (OR= 2.9, p = 0.040, 95%CI= 1.1-7.8) compared to those who did not suffer from hunger at increased risk of depleted body iron stores. We also found participants who were anaemic were more likely to suffer from both severe hunger and depleted body iron stores, suggesting a combined effect. This implies that severe maternal hunger could have caused depleted body iron stores, leading to the risk of anaemia. Although we observed that malaria tablets received at ANC effectively determined haemoglobin levels of the participant, those who were given tended to be more anaemic, implying that that malaria was not the likely cause of iron deficiency anaemia among the teenagers.

The body requires iron, vitamin B_{12}, and folate for the effective production of haemoglobin. Any deficiency in these micronutrients are likely to cause anaemia due to inadequate production of red blood cells. Poor dietary intake from inadequacies of micronutrients and poor dietary diversity are the main culprit for low vitamin and mineral levels, and therefore anaemia [25, 63]. We observed that teenagers who had inadequate women's dietary diversity score (WDDS) compared to those with adequate WDDS were more likely to have a depleted body iron stores (p= 0.046), and that, inadequate WDDS among the teenagers significantly contributed to poorer iron stores and anaemia occurrence, but the regression model showed a lower risk for depleted body iron stores (OR= 0.3, p = 0.089, 95%CI= 0.1-1.2). We also found that more

pregnant teenagers with depleted body iron stores had inadequate multiple micronutrient intakes than those with normal body iron stores (p = 0.046), and that, inadequate multiple micronutrient intakes (iron-folate-vitamin B_{12}) contributed to depleted body iron stores and anaemia occurrence in the teenagers. This implies that consumption of less diversified diet together with multiple micronutrient inadequacies during pregnancy is associated with depleted iron stores, and can consequently lead to maternal anaemia. A study by Diana et al. [64] in Indonesia reported the consumption of a less diversified diet among pregnant, which was not associated with iron inadequacy, contributed to anaemia in pregnant women. Hence, supplementation of iron and folic acid to pregnant women need to be re-evaluated to factor the additional demands of adolescent girls, and those who might be at risk of iron deficiency anaemia. Also, appropriate nutrition education at the antenatal care clinics should focus on educating pregnant women to consume nutritious and diversified diets, to improve the nutrition status of iron.

Most often, women in low-and-middle-income countries go into pregnancy malnourished, and the demands of gravidity can worsen micronutrient deficiencies, leading to iron deficiency anaemia. In Ghana, Nonterah et al. [32] studying both pregnant teenagers and adults reported multiparity and maternal underweight as predictors of anaemia. In this study maternal BMI was not associated with iron deficiency anaemia, although underweight prevalence was higher among anaemic teenagers compared to non-anaemic participants. On the other hand, the regression analysis showed that participants who were wasted (using MUAC) (OR= 1.2, p = 0.543, 95%CI= 0.6-2.3) were more likely to be anaemic, and this was consistent with a study among pregnant adults in Ghana [26].

We observed that being depleted of body iron stores compared to normal body iron stores was associated with an increased risk of anaemia. Deficient systemic supply of iron (high ZPP/Hb ratio; (OR= 9.7, p<0.001, 95%CI= 6.0-15.8),) and depleted body iron stores (OR= 3.0, p = 0.167, 95%CI= 0.6-14.6) during pregnancy were both predictors of iron deficiency anaemia in the teenagers, with an odds ratio greater than 2. Participants with a deficient supply of iron were more likely to have poorer iron stores and become

anaemic. Similarly, participants with low serum ferritin levels were more likely to have poorer iron stores and become anaemic. These imply that low serum ferritin and deficient systemic supply of iron could also be associated with depleted iron stores in the teenagers, thus lead to the occurrence of maternal anaemia. A depletion of iron stores occurs when there is a poor dietary intake, which affects the systemic supply of iron for haemoglobin production [25, 62]. In the body, ZPP and serum ferritin are among iron biomarkers that affect iron status, and the combination of the two can predict whether iron stores are depleted or not [55]. Ferritin is the main body storage of iron, and it can be elevated (above 200 µg/L) during inflammation state, infection, which causes iron overload [49, 55]. Wirth et al. [9] reported maternal inflammation, vitamin A insufficiency, but not malaria, vitamin $B_{12,}$ and folate deficiencies to significantly determine maternal anaemia. In this study, ferritin levels were less than 200µg/L for the population, which depicts no likely inflammation or infection. Where there is no inflammation, the occurrence of low serum ferritin, and deficient ZPP/Hb ratio depict poor dietary intakes and poor iron bioavailability [49]. Also, the final step of haemoglobin production involves a combination of ferrous protoporphyrin and globin, but where iron is deficient, iron is replaced by zinc to produce zinc protoporphyrin (ZPP) in a normal ratio 30,000:1. But progressive iron deficiency increases ZPP levels, which has a profound effect on the supply of iron for haemoglobin production [48, 66]. In this study, we observed a high ZPP/Hb ratio among the teenagers who were anaemic, indicating deficient iron supply for haemoglobin production. All these associations tend to explain that poor micronutrient intake and severe hunger, and low serum ferritin could contribute to deficient systemic iron supply and concurrent depleted body iron stores, leading to iron deficiency anaemia among pregnant teenagers.

The implication of this study is; in our previous study, we reported that a higher prevalence of anemia among pregnant teenagers than adults, and mean Hb levels of both teenagers and adults were found to be higher by the 36th week of pregnancy than at antenatal registration. Also, a positive correlation was observed between Hb at antenatal registration and 36th-week gestation, indicating that pre-pregnancy

Hb levels, largely determined anaemia occurrence during the latter stages of pregnancy [21]. A follow-up study was needed to understand the associated factors causing high anaemia among teenagers than adults. And since most studies in Ghana had focused on pregnant adults, the teenagers who were at greater risk of anaemia were considered for the current study. The current findings have provided enough evidence that the high maternal anaemia prevalence among teenagers was a result of hunger experienced during pregnancy, inadequate multiple micronutrient intakes, low serum ferritin levels which contributed to the deficient systemic supply of iron and depleted body iron stores. Other factors such as wasting and inadequate dietary diversity contributed to depleted iron stores, and anaemia occurrence. Additionally, malaria tablets given at antenatal clinic, MUAC, ZPP, ZPP/Hb ratio, and body iron stores ($p<0.001$) had a significant effect on the Hb levels of teenagers. Malaria tablet given at ANC affected Hb levels but was not a significant contributor of iron deficiency anaemia in the teenagers. There is therefore the need for nutrition education and women empowerment among these teenagers, and also antenatal service delivery should comprehensively focus on the nutritional needs of pregnant women, while special attention should be given to teenagers to prevent the devastating consequences of anaemia during pregnancy. Also, interventions that encourage food fortification and diversification has been reported to reduce anaemia in women of child-bearing age, and these interventions can be considered [67].

Conclusions

Iron deficiency anaemia was found among pregnant teenagers in this study. Iron deficiency anaemia is common among pregnant teenagers and the related factors include malaria tablets given at ANC, maternal hunger, maternal MUAC, a deficient systemic supply of iron, and depleted body iron stores. Antenatal service delivery should focus on the nutritional needs of pregnant women, while special attention is given to teenagers to prevent the devastating consequences of anaemia during pregnancy.

Acknowledgments

We are grateful for the support received from the directors of health, health workers in the health centers where the study took place, and the pregnant adolescents.

References

1. Dahl RE, Allen NB, Wilbrecht L, Suleiman AB. Importance of investing in adolescence from a developmental science perspective. Nature. 2018; 554: 441–50.
2. Nguyen PH, Scott S, Neupane S, Tran LM. and Menon P. Social, biological, and programmatic factors linking adolescent pregnancy and early childhood undernutrition: a path analysis of India's 2016 National Family and Health Survey. Lancet Child Adolecent Health. 2019; 3: 463-473. http://dx.doi.org/10.1016/S2352-4642(19)30110-5.
3. Yussif A, Lassey A, Ganyaglo GY, et al. The long-term effects of adolescent pregnancies in a community in Northern Ghana on subsequent pregnancies and births of the young mothers. Reprod Health. 2017; 14: 178. https://doi.org/10.1186/s12978-017-0443-x.
4. World Health Organization. Adolescent pregnancy. 2019a. Available at https://apps.who.int/iris/bitstream/handle/10665/329883/WHO-RHR-19.15-eng.pdf. Accessed 5 June 2020.
5. Ghana Statistical Service (GSS), Ghana Health Service (GHS), ICF Macro. Ghana Demographic and Health Survey. GSS, GHS, & ICF Macro, Accra (2014).
6. Neal S, Matthews Z, Frost M, et al. Childbearing in adolescents aged 12–15 years in low resource countries: a neglected issue. New estimates from demographic and household surveys in 42 countries. Acta Obstet Gynecol Scand. 2012; 91: 1114–18.
7. Azevedo WF, Diniz MB, Fonseca ES, Azevedo LM. & Evangelista CB. Complications in adolescent pregnancy: Systematic review of the literature. Einstein (Sao Paulo, Brazil). 2015; 13(4): 618–626.

8. WHO. Global Health Estimates 2015: deaths by cause, age, sex, by country and by region, 2000-2015. Geneva, World Health Organization, 2016.
9. Wirth JP, Woodruff BA, Engle-Stone R, Namaste SML, et al. Predictors of anemia in women of reproductive age: Biomarkers Reflecting Inflammation and Nutritional Determinants of Anemia (BRINDA) project. Am J Clin Nutr. 2017;106 (Suppl):416S–27S. doi: https://doi.org/10.3945/ajcn.116.143073.
10. Lumor O, Dzabeng F. and Adanu RM. Factors influencing the use of anemia preventing measures among antenatal clinic attendees in the Kintampo North Municipality, Ghana. Afr J Reprod Health. 2019; 23(2): 35-43.
11. Sheehan P, Sweeny K, Rasmussen B, et al. Building the foundations for sustainable development: a case for global investment in the capabilities of adolescents. Lancet. 2017; 390: 1792–806.
12. Means R.T. Iron deficiency and iron deficiency anemia: implications and impact in pregnancy, fetal development, and early childhood parameters. Nutrients. 2020; 12: 447. doi:10.3390/nu12020447.
13. Mettananda S, Suranjan M, Fernando R, Dias T, Mettananda C, Rodrigo R, et al. Anaemia among females in child-bearing age: Relative contributions, effects and interactions of alpha- and beta thalassaemia. PLoS ONE. 2018, 13, e0206928.
14. Chen C, Grewal J, Betran AP, Vogel JP, Souza JP. and Zhang J. Severe anemia, sickle cell disease, and thalassemia as risk factors for hypertensive disorders in pregnancy in developing countries. Pregnancy Hypertens. 2018; 13: 141–147.
15. World Health Organization. Nutritional anaemias: tools for effective prevention and control. Geneva: World Health Organization; 2017; Available at: https://apps.who.int/iris/rest/bitstreams/1091289/retrieve.pdf. Accessed 4 June 2020.

16. WHO. Intermittent iron and folic acid supplementation during pregnancy. 2019b. Available at https://www.who.int/elena/titles/intermittent_iron_pregnancy/en/. Accessed 5 June 2020.

17. Von Look P, Lincetto O, Fogstad H, et al. Iron and folate supplementation: Integrated management of pregnancy and childbirth (IMPAC). Geneva: World Health Organization; 2006.

18. Zerfu TA, Ayele HT. Micronutrients and pregnancy; effect of supplementation on pregnancy and pregnancy outcomes: A systematic review. Nutr J. 2013;12(1):20. https://doi.org/10.1186/1475-2891-12-20.

19. Ministry of Health. Guidelines for malaria in pregnancy. Accra: Ministry of Health, Ghana; 2014.

20. University of Ghana, GroundWork, University of Wisconsin-Madison, KEMRI-Wellcome Trust, UNICEF. Ghana Micronutrients Survey 2017. Accra, Ghana; 2017.

21. Ampiah MKM, Kovey JJ, Apprey C. and Annan RA. Comparative analysis of trends and determinants of anaemia between adult and teenage pregnant women in two rural districts of Ghana. BMC Public Health. 2019; 19: 1379. https://doi.org/10.1186/s12889-019-7603-6.

22. Huch R Sr. Iron defi ciency and iron deficiency anaemia. Stuttgart: Thieme, 2006.

23. Lopez A, Cacoub P, Macdougall IC. And Peyrin-Biroulet L. Iron deficiency anaemia. Lancet. 2016; 387: 907-916. http://dx.doi.org/10.1016/S0140-6736(15)60865-0.

24. Kirkpatrick SI. and Tarasuk V. Food insecurity is associated with nutrient inadequacies among Canadian adults and adolescents. J. Nutr. 2008; 138:604–612. doi: 10.1093/jn/138.3.604.

25. Abbaspour N, Hurrell NA. and Kelishadi R. Review on iron and its importance for human health. J Res Med Sci. 2014; 19(2): 164-174.

26. Agbozo F, Abubakari A, Der J. and Jahn A. Maternal dietary intakes, red blood cell indices and risk for anemia in the first, second and third trimesters of pregnancy and at predelivery. Nutrients. 2020; 12: 777; doi:10.3390/nu12030777.

27. Pobee RA, Aguree S, Colecraft EK, Gernand AD. and Murray-Kolb LE. Food Insecurity and Micronutrient status among Ghanaian women planning to become pregnant. Nutrients. 2020; 12: 470; doi:10.3390/nu12020470.
28. Baidoo SE, Tay SCK, Obiri-Danso K, Abruquah HH. Intestinal helminth infection and anaemia during pregnancy: A community-based study in Ghana. J Bacteriol Res. 2010;2(2):9–13.
29. Apea-Kubi KA, Yamaguchi S, Sakyi B, Kishimoto T, Ofori-Adjei D, Hagiwara T. Neisseria gonorrhoea, Chlamydia trachomatis, and Treponema pallidum infection in antenatal and gynecological patients at Korle-Bu teaching hospital, Ghana. J Infect Dis. 2004;57(6):252–256.
30. Völker F, Cooper P, Bader O, et al. Prevalence of pregnancy-relevant infections in a rural setting of Ghana. BMC Pregnancy Childbirth. 2017;17(1):172. https://doi.org/10.1186/s12884-017-1351-3.
31. Engmann C, Adanu R, Lu T-S, Bose C, Lozoff B. Anemia and iron deficiency in pregnant Ghanaian women from urban areas. Int J Gynecol Obstet. 2008;101(1):62–66. https://doi.org/10.1016/j.ijgo.2007.09.032.
32. Nonterah EA, Adomolga E, Yidana A, Kagura J, Agorinya I, et al. Descriptive epidemiology of anaemia among pregnant women initiating antenatal care in rural Northern Ghana. African Journal of Primary Health Care & Family Medicine. 2019; 11(1): a1892. https://doi.org/10.4102/phcfm.v11i1.1892.
33. Wemakor A. Prevalence and determinants of anaemia in pregnant women receiving antenatal care at a tertiary referral hospital in Northern Ghana. BMC Pregnancy and Childbirth. 2019; 19: 45. https://doi.org/10.1186/s12884-019-2644-5.
34. Anlaaku P. and Anto F. Anaemia in pregnancy and associated factors: a cross sectional study of antenatal attendants at the Sunyani Municipal Hospital, Ghana. BMC Res Notes. 2017; 10: 402. DOI 10.1186/s13104-017-2742-2.

35. Ghana Statistical Service. Population by region. 2019. Retrieved 24 May 2020, from http://www.statsghana.gov.gh/regionalpopulation.php?population=MTI5MzE3OTU5OC40NDg1&&Ashanti&regid=1.
36. Ghana Statistical Service (GSS), Ghana Health Service (GHS), and ICF. Ghana Maternal Health Survey 2017: Key Indicators Report. 2018. Accra, Ghana: GSS, GHS, and ICF. Available online at http://www2.statsghana.gov.gh/docfiles/PR95.pdf. Accessed 20 May 2020.
37. Ayensu J, Edusei A, Oduro I. & Larbie C. Status of some antioxidant micronutrient and pregnancy outcomes in Ghanaian status of some antioxidant micronutrient and pregnancy outcomes in Ghanaian Adolescents Attending Antenatal Clinic in Urban (Suntreso) and Rural (Mampong) Hospitals. EJNFS. 2017; 7(2): 120–127. https://doi.org/10.9734/EJNFS/2017/24209.
38. Food Science and Nutrition Department, University of Ghana. The nutrient analysis template software excel spreadsheet for Ghanaian foods, 2010.
39. National Academy of Sciences, Food and Nutrition Board, Institute of Medicine. Dietary reference intakes series. Washington, DC: National Academies Press, 2005. in text ‘Mahan, L.K., Escott-Stump, S. and Raymond, J.L. (2012). Krause’s Food and the Nutrition Care Process, 13th Editions, Elsevier Saunders Publication, Missouri, USA, page 1.
40. FAO and FHI. Minimum Dietary Diversity for Women A Guide to Measurement. 2016.
41. Kennedy G, Ballard T, Dop M. Guidelines for measuring household and individual dietary diversity. FAO. 2010.
42. Ballard, Terri, Deitchler JCASM. Household Hunger Scale : Indicator Definition and. Nutrition. 2011;(August).
43. Mattes, Robert. Afrobarometer; The material and political bases of lived poverty in Africa:20 insights from the Afrobarometer. 2008.

44. WHO expert consultation. Appropriate body mass index for Asian populations and its implications for policy and intervention strategies. The Lancet. 2004; 157-163.

45. Lopez LB, Calvo E, Poy M, del Valle Balmaceda Y, Camera K. Changes in skinfolds and mid-upper arm circumference during pregnancy in Argentine women. Matern Child Nutr. 2011; 7(3):253-262. https:// doi.org/10.1111/j.1740-8709.2009.00237.x

46. Fakier A, Petro G. and Fawcus S. Mid-upper arm circumference: A surrogate for body mass index in pregnant women. S Afr Med J. 2017;107(7):606-610. DOI:10.7196/SAMJ.2017.vi7.12255.

47. World Health Organization (WHO). Maternal anthropometry and pregnancy outcomes. Bull World Health Organ. 1995, 73(suppl 1).

48. World Health Organization, Centers for Disease Control and Prevention. Assessing the iron status of populations. 2nd ed. 2007. http://apps.who.int/iris/bitstream/10665/75368/1/9789241596107_eng.pdf?ua=1. Accessed 18 June 2020.

49. Dignass A, Farrag K, Stein J. Limitations of Serum Ferritin in Diagnosing Iron Deficiency inInflammatory Conditions. International Journal of Chronic Diseases. 2018; https://doi.org/10.1155/2018/9394060.

50. Benzie I.F and Strain JJ. The Ferric Reducing Ability of Plasma (FRAP) as a Measure of "Antioxidant Power": The FRAP Assay. Annal Biochem. 1996; 239(1): 70-6. doi: 10.1006/abio.1996.0292

51. WHO. Serum Ferritin Concentrations for The Assessment of Iron Status and Iron Deficiency in Populations. Vitamin and Mineral Nutrition Information System. Geneva: World Health Organization; 2011.

52. Ozturk G, Ozgu-Erdinc AS, Ucar F, Ginis Z, Erden G. and Danisman N. Concentration of prealbumin and some appetite controlling hormones in pregnancies associated with hyperemesis gravidarum. Annals of Biochemistry. 2017; 54(2): 258-263. Doi.10.1177/0004563216654724.

53. Stanton NV, Gunter EW, Parsons PJ, Field PH. Empirically determined lead- poisoning screening cutoff for the Protofluor-Z hematofluorometer. Clin Chem. 1989; 35: 2104-7.

54. Yu KH. Effectiveness of zinc protoporphyrin/heme ratio for screening iron deficiency in preschool-aged children. Nutrition Research and Practice. 2011; 5(1): 40-45. DOI: 10.4162/nrp.2011.5.1.40.

55. Labbe RF, Vreman HJ, Stevenson DK. Zinc protoporphyrin: A metabolite with a mission. Clinical Chemistry. 1999; 45:2060–2072.

56. Ghana Statistical Service (GSS), Ghana Health Service (GHS), ICF International. Demographic and health survey 2014. Rockville, MD: GSS, GHS, and ICF International; 2015.

57. Ayensu J, Annan R, Lutterodt H, Edusei A, Peng L. Prevalence of anaemia and low intake of dietary nutrients in pregnant women living in rural and urban areas in the Ashanti region of Ghana. PLoS ONE. 2020; 15(1): e0226026. https://doi.org/10.1371/journal.pone.0226026.

58. Srour MA, Aqel SS, Srour KM, Younis KR. and Samarah F. Prevalence of anemia and iron deficiency among Palestinian pregnant women and its association with pregnancy outcome. Anemia. 2018; https://doi.org/10.1155/2018/9135625.

59. Obai G, Odongo P. and Wanyama R. Prevalence of anaemia and associated risk factors among pregnant women attending antenatal care in Gulu and Hoima Regional Hospitals in Uganda: A cross-sectional study. BMC Pregnancy and Childbirth. 2016; 16:76. DOI 10.1186/s12884-016-0865-4.

60. Al-Mehaisen L, Khader Y, Al-Kuran O, Abu F. and Amarin Z. Maternal anemia in rural Jordan: Room for improvement. Anemia. 2011; vol. 2011, Article ID 381812.

61. Tarasuk VS. Household food insecurity with hunger is associated with women's food intakes, health and household circumstances. J. Nutr. 2001; 131: 2670–2676. doi: 10.1093/jn/131.10.2670.

62. De Benoist B, McLean E, Egli I, Cogswell M, editors. Geneva: WHO Press, World Health Organization; 2008.
63. Chaparro CM. and Suchdev PS. Anaemia epidemiology, pathophysiology, and etiology in low-and-middle-income countries. Ann N Y Acad Sci. 2019; 1450(1): 15–31. doi:10.1111/nyas.14092.
64. Diana R, Khomsan A, Anwar F, Christianti DF, Kusuma R. and Rachmayanti RD. Dietary quantity and diversity among anaemic pregnant women in Madura Island, Indonesia. Journal of Nutrition and Metabolism. Vol 2019; 1-7. https://doi.org/10.1155/2019/2647230.
65. Gernand AD, Schulze KJ, Stewart CP. and West KP. Micronutrients deficiencies in pregnancy worldwide. Health effects and prevention. Nature Review Endocrinology. 2016; 12(5) DOI: 10.1038/nrendo.2016.37.
66. Peng YY. and Uprichard J. Ferritin and iron studies in anaemia and chronic disease. Annals of Clinical Biochemistry: International Journal of Laboratory Medicine. 2017; 54(1): 43-48. https://doi.org/10.1177/0004563216675185.
67. Awaluddin SM, Ahmad NA, Naidu BM, Mohamad MS, Yusof M, et al. A population-based anaemia screening using point-of care in estimating prevalence of anaemia in Malaysian adults: findings from a Nationwide survey. Journal of Community Medicine and Health Education. 2017; 7: 513.

Figure

Click here to access/download;Figure;Fig. 1. tiff.tiff

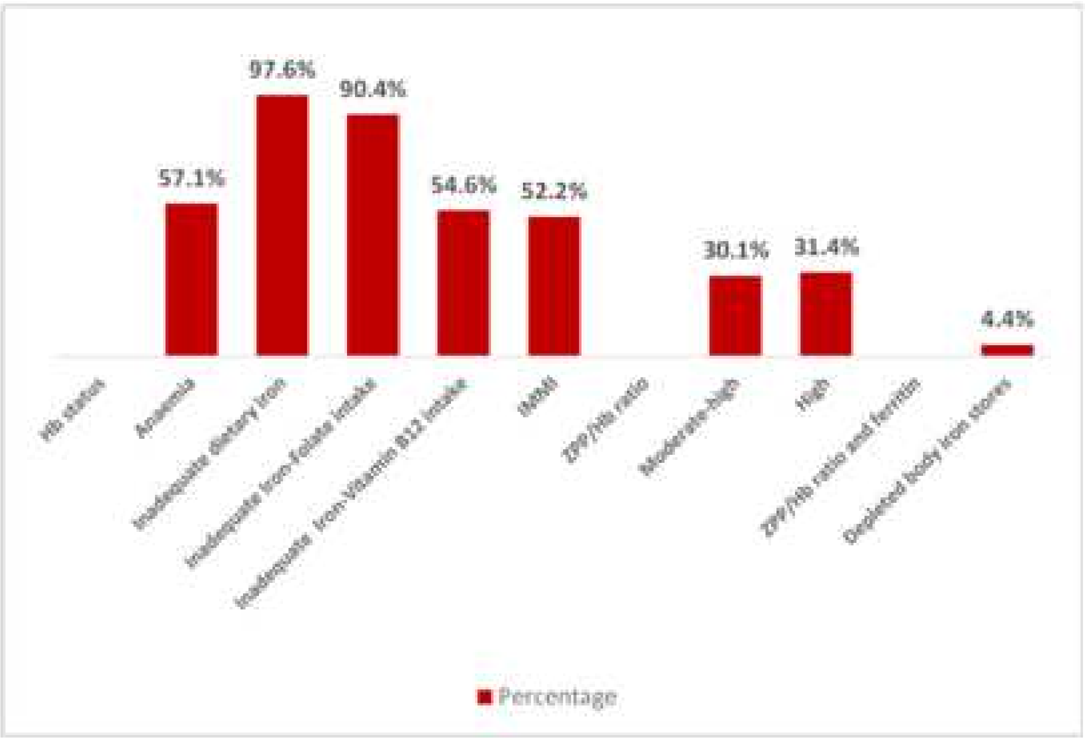

Hb- Haemoglobin, ZPP- Zinc protoporphyrin, IMMI- Inadequate Multiple Micronutrient Intakes for dietary iron, folate and vitamin B_{12}

Fig 1. Iron-deficiency anaemia prevalence among pregnant teenagers
