# Supplementary material for: Factors associated with iron deficiency anaemia among pregnant teenagers in Ashanti Region, Ghana: A hospital-based prospective cohort study
Source: PLoS One. 2021 Apr 27;16(4):e0250246. doi: 10.1371/journal.pone.0250246 (PMC8078754; doi:10.1371/journal.pone.0250246)
Supplement: Attachment — Submitted filename: Review_of_PONE.docx [file pone.0250246.s004.docx]

1. Describe the formula used to estimate the sample size of 420.

2. If Non Parametric test has been used (Mann Whitney U test), then continuous variables may be expressed as Median and Inter quartile range.

3. For variables such as mean MUAC, mean Serum PAB, mean Serum Feeritin, it seems that Independent t test is appropriate rather than Mann Whitney U test as mentioned in Table 2 and Table3.

4.Mention the quality and adequacy of the model used.
