# Supplementary material for: Factors associated with iron deficiency anaemia among pregnant teenagers in Ashanti Region, Ghana: A hospital-based prospective cohort study
Source: PLoS One. 2021 Apr 27;16(4):e0250246. doi: 10.1371/journal.pone.0250246 (PMC8078754; doi:10.1371/journal.pone.0250246)
Supplement: Attachment — Submitted filename: Second_revision_Response to reviewers.docx [file pone.0250246.s007.docx]

Department of Biochemistry and Biotechnology

College of Science, KNUST,

Kumasi, Ghana.

Joerg Heber, *Ph.D*.,

The Editor-in-Chief,

PLoS One Corporation,

California, USA.

**POINT-BY-POINT REVISIONS IN RESPONSE TO REVIEWER/EDITOR COMMENTS**

Dear Editor-in-Chief,

Thanks for reviewing our manuscript.

In line with the reviewer’s comments, the following revisions have been made to the Manuscript ID: PONE-D-20-21782R1 entitled “**Factors associated with Iron Deficiency Anaemia among pregnant teenagers in Ghana:** **A hospital-based prospective cohort study’** by **Reginald Adjetey Annan, Linda Afriyie Gyimah, Charles Apprey, Anthony Kwaku Edusei, Odeafo Asamoah-Boakye, Linda Nana Esi Aduku, Wisdom Azanu, and Herman E. Lutterodt**. Below is our submission, highlight in **blue colour**, and yellow in the revised manuscript.

Journal Requirement:

1. Please review your reference list to ensure that it is complete and correct. If you have cited papers that have been retracted, please include the rationale for doing so in the manuscript text, or remove these references and replace them with relevant current references. Any changes to the reference list should be mentioned in the rebuttal letter that accompanies your revised manuscript. If you need to cite a retracted article, indicate the article’s retracted status in the References list and also include a citation and full reference for the retraction notice

Response: The reference list has been revised and it is complete. All references are correctly cited and none have been retracted. However, we have added another reference in our discussion and made changes to the numbering of the references. Please see the highlighted yellow in the discussion and reference list as the new changes made. Below is the new cited reference:

63.	Gyimah LA, Annan RA, Apprey C, Edusei A, Aduku LNE, Asamoah-Boakye O, Azanu W. and Lutterodt H. Dietary diversity and its correlates among pregnant adolescent girls in Ghana. PLoS ONE. 2021; 16(3): e0247979. https://doi.org/10.1371/journal.pone.0247979.

Response to Academic Editor Comment

Point 1: However, the figure presented lacks few pre-requisites for a bar chart. It has been pointed out by a reviewer and even after that the revised figure does not have Y axis and there are errors. It's suggested that you can think of presenting the data in the figure in a different format and submit a revised version of the manuscript.

Response 1: Figure 1 has been revised to show Y axis and errors addressed. Also,the figure was passed through PACE for the correct ‘tif‘ figure format of Plos One. Please see figure 1 file.

Yours faithfully,

Dr. Reginald Adjetey Annan.

(Corresponding Author).
